# Use of a New International Classification of Health Interventions for Capturing Information on Health Interventions Relevant to People with Disabilities

**DOI:** 10.3390/ijerph15010145

**Published:** 2018-01-17

**Authors:** Nicola Fortune, Richard Madden, Ann-Helene Almborg

**Affiliations:** 1 National Centre for Classification in Health, University of Sydney, Camperdown, NSW 2006, Australia; richard.madden@sydney.edu.au; 2 National Board of Health and Welfare, SE-10630 Stockholm, Sweden; ann-helene.almborg@socialstyrelsen.se; 3 Nordic WHO Family of International Classifications Collaborating Centre, Directorate for E-Health, NO-0130 Oslo, Norway

**Keywords:** health promotion, rehabilitation, people with disabilities, health intervention, classification, International Classification of Health Interventions (ICHI)

## Abstract

Development of the World Health Organization’s International Classification of Health Interventions (ICHI) is currently underway. Once finalised, ICHI will provide a standard basis for collecting, aggregating, analysing, and comparing data on health interventions across all sectors of the health system. In this paper, we introduce the classification, describing its underlying tri-axial structure, organisation and content. We then discuss the potential value of ICHI for capturing information on met and unmet need for health interventions relevant to people with a disability, with a particular focus on interventions to support functioning and health promotion interventions. Early experiences of use of the Swedish National Classification of Social Care Interventions and Activities, which is based closely on ICHI, illustrate the value of a standard classification to support practice and collect statistical data. Testing of the ICHI beta version in a wide range of countries and contexts is now needed so that improvements can be made before it is finalised. Input from those with an interest in the health of people with disabilities and health promotion more broadly is welcomed.

## 1. Introduction

Health systems provide a wide range of services relevant to supporting the health of people with disabilities. Health interventions spanning primary, secondary and tertiary prevention, and curative and rehabilitative interventions, are of as much importance for people with disabilities as they are for the population more broadly. Interventions that aim to restore, maintain or support functioning, and to optimise participation across all life domains, whether they are provided over a defined time period or are ongoing, are of particular relevance for people with disabilities.

Information about health interventions is needed to support health system function [1]. In particular, information about interventions provided and received is required to assess quality of services, accessibility, equity of utilisation, and overall efficiency of the system. Understanding the extent to which services are meeting the needs of different groups within a population informs improved health system planning and resourcing, towards the ultimate objective of equity in health. Being able to collect consistent, comparable data on health interventions is essential for this. 

International standard classifications provide a solid basis for collecting, aggregating, analysing, and comparing the data that are needed to inform the activities of health systems, and are used in a wide range of data capture mechanisms including administrative databases, surveys, and electronic health records [2,3,4]. Standard classifications also provide a common language and common conceptual structures to aid communication within and between communities of practice, and between different components of health and human services systems [5]. Development of the World Health Organization’s International Classification of Health Interventions (ICHI) began in 2007. In 2017 ICHI was released as a beta version for testing [6]. The development of ICHI is an international project undertaken through the network of collaborating centres around the world that assists WHO in developing and maintaining its family of health classifications; the work has involved input from a wide range of experts. Once it is finalised, ICHI will sit alongside the International Classification of Diseases (ICD) [7] and the International Classification of Functioning, Disability and Health (ICF) [8] as a member of the WHO’s Family of International Classifications [4]. Together, the three classifications provide a comprehensive toolkit for capturing information about health conditions, functioning, and the interventions delivered to support and promote the health of individuals and populations. 

This new classification is of particular significance, as it is designed to describe the full range of interventions needed to improve health. Health in ICHI is defined in the same broad way as in the ICF, following the bio-psychosocial model. Good health involves not just preventing and treating disease, but also the achievement of optimal functioning. With this broad definition of health, ICHI will allow a consistent view of all health interventions relevant to people with disabilities, including interventions that target aspects of the environment in which people live. 

In this paper, we first give an overview of ICHI, then discuss the ways in which it will provide a basis for capturing information on the different types of health interventions relevant to people with disabilities, including interventions to support functioning and both individual and population level health promotion interventions. To illustrate the value of a standard health interventions classification, we describe early experiences of the use of the Swedish National Classification of Social Care Interventions and Activities (KSI), modelled on ICHI, to support practice and collect statistical data. Finally, we invite readers to have input into the testing and further development of ICHI.

## 2. The International Classification of Health Interventions (ICHI)

A health intervention as defined in ICHI is ‘an act performed for, with or on behalf of a person or a population whose purpose is to assess, improve, maintain, promote or modify health, functioning or health conditions’. The broad coverage of ICHI flows from this definition. The classification encompasses interventions delivered across all functional sectors of the health system—medical, surgical, primary care, community health, rehabilitation, allied health, mental health, nursing, assistance with functioning and public health [6]. 

**Figure 1:**
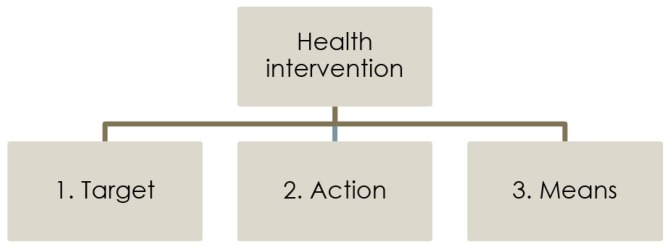
The three axes to describe a health intervention in ICHI.

ICHI is built around three axes (Figure 1):

- Target: the entity on which the Action is carried out;
- Action: the deed done by an actor to the Target;
- Means: the processes and methods by which the Action is carried out.

Each axis is a coded list of descriptive categories, and each intervention is represented by a title and a unique seven-character code denoting the Target, Action and Means for that intervention. Each ICHI intervention has a unique combination of categories from the three axes [6].

ICHI provides comprehensive coverage of medical and surgical interventions, like the many national intervention classifications that now exist; the targets for these interventions are the anatomy. Additionally, the full range of Body Functions, Activities and Participation domains and Environmental Factors from the ICF are included as targets in ICHI. These are used to describe investigative interventions, interventions to improve the functioning of body systems, interventions to support people in activities and participation, and interventions to ameliorate environmental barriers for people with a disability. There is also a list of Health-related Behaviour targets. Environmental Factor and Health-related Behaviour targets are used in ICHI to describe public health and health promotion interventions. 

The classification consists of 27 chapters grouped into four sections:

1. Interventions on Body Systems and Functions (Chapters 1–12)
2. Interventions on Activities and Participation Domains (Chapters 13–21)
3. Interventions on the Environment (Chapters 22–26)
4. Interventions on Health-related Behaviours (Chapter 27)

More information about an intervention can be recorded by adding extension codes to the ICHI intervention code. ICHI extension codes include lists of assistive products, therapeutic products and medicaments, to use where relevant, and codes for recording a range of other additional descriptive information. Intervention codes can be used as building blocks to describe the complex, multifaceted interventions that are characteristic of health promotion, or packages of interventions delivered to an individual or a group, such as a rehabilitation program [6]. Coding rules and examples to illustrate use of ICHI intervention codes and extension codes are included in the ‘guidelines for users’, available in the online version of the draft classification.

ICHI is designed primarily for use as a statistical classification, to provide a stable and agreed basis for the production of summary data. As stated in the introduction to the classification, information about the reason(s) for an intervention or its outcome should be classified using ICD and ICF. In any given application, other data items or information fields can be used alongside ICHI codes, as needed, to capture other information that may be required for describing interventions, such as who provides the intervention and where it is performed, aspects of intervention context, and measures of duration, intensity or dose [9,10]. In the introduction to ICHI it is anticipated that countries may develop extended versions of the classification to meet national-level needs by adding more detail.

## 3. People with Disability and Health Interventions

The full range of interventions provided by health systems is relevant to people with disabilities. The vision articulated in the WHO Global Disability Action Plan 2014–2021 is ‘a world in which all persons with disabilities and their families live in dignity, with equal rights and opportunities, and are able to achieve their full potential’ [11] (p. 2). Access to health services is known to be a particular issue for people with disabilities, with a range of environmental barriers preventing equitable access [12]. As stated in the Plan, ‘disability is a global public health issue because people with disability, throughout the life course, face widespread barriers in accessing health and related services’ [11] (p. 1). The Plan sets out three objectives:

(1) to remove barriers and improve access to health services and programmes;
(2) to strengthen and extend rehabilitation, habilitation, assistive technology, assistance and support services, and community-based rehabilitation; and
(3) to strengthen collection of relevant and internationally comparable data on disability and support research on disability and related services’ [11] (p. 2).

Access to health services is the subject of Article 25 of the United Nations Convention on the Rights of Persons With Disabilities, which calls on Parties to “recognize that persons with disabilities have the right to the enjoyment of the highest attainable standard of health”, and to ensure that persons with disabilities are provided “the same range, quality and standard of free or affordable health care and programs as provided to other persons” and are provided “those health services needed by persons with disabilities specifically because of their disabilities” [13] (p. 18). Further, Article 31 of the Convention requires that “States Parties undertake to collect appropriate information, including statistical and research data, to enable them to formulate and implement policies to give effect to the present Convention” [13] (p. 23).

ICHI can provide a basis for capturing data on met and unmet need for specific health interventions among the population with a disability. Such data are needed for advocacy on this issue, and to inform improved practice and policy. Below we bring a focus to the potential utility of ICHI for collecting information on and facilitating communication about interventions to support functioning and health promotion interventions for people with a disability.

### 3.1. Interventions to Support Functioning

As defined in the ICF, “Activity is the execution of a task or action by an individual” and “Participation is involvement in a life situation” [8] (p. 10). People with disabilities may require a range of services and supports to improve or maintain body functions, and to assist them in activities and participation. Interventions may involve removing environmental barriers or putting environmental facilitators in place. Examples of ICHI interventions targeting body functions, activities and participation domains and environmental factors are given in Table 1, showing the Target, Action and Means for each intervention.

Type of assistive product can be specified, where relevant, using an extension code appended to the intervention code, for example “Communication boards/books/cards” (XP330.01). The list of assistive products in ICHI includes all assistive products in WHO’s Priority Assistive Products List [14,15]. 

The use of ICF domains as targets for interventions in ICHI means that these two classifications can readily be used together. ICF can describe a person’s functioning, functioning-related goals and need for assistance, and ICHI can be used to describe interventions delivered. Further, ICF can be used to look at how a person’s functioning is affected by the intervention/s provided (i.e., by comparing the person’s ICF functioning profile before and after the intervention). Where necessary, ICD codes can record any relevant information about health conditions. 

**Figure 2:**
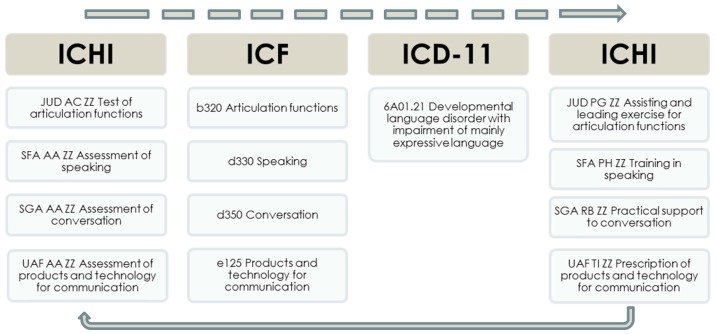
Using the three WHO classifications together—example of coding for interventions, level of functioning, functioning goals and diagnosis relevant to a person with communication-related functioning limitations.

Application of the three WHO health classifications together is illustrated in Figure 2, showing codes that may be recorded for a person with communication-related functioning limitations. ICHI is used to record the initial investigative interventions conducted, then ICF is used to describe the person’s functioning in relevant body functions and activities and participation domains and to note the need for environmental facilitators (“Products and technology for communication”); an ICD diagnosis code is also recorded. ICF may also be used to document functioning-related goals agreed with the person. ICHI is then used to record the therapeutic and supportive interventions delivered to address the person’s goals. At follow-up, ICHI is again used to record investigative interventions conducted, and ICF to describe the person’s functioning, which can be compared with their pervious ICF functioning profile and used for assessing the level of achievement of the functioning-related goals previously set [16,17].

Interventions focused on changing environmental facilitators or barriers that affect functioning can be recorded with ICHI intervention codes that use ICF Environmental Factors categories as targets. These include codes such as “Environment modification to influence light” (UBH TM ZZ), “Environment modification to buildings for public use” (UAK TM ZZ), “Provision of products and technology for personal use in daily living” (UAD RD ZZ), “Training in the use of products and technology for personal indoor and outdoor mobility and transportation” (UAE PH ZZ), “Deconditioning from products and technology for communication” (UAF PJ ZZ), and “Advising about products and technology for employment” (UAH PN ZZ).

### 3.2. Health Promotion Interventions

The Rio Political Declaration on Social Determinants of Health reaffirmed equity in health as a fundamental principle in health promotion [18]. It is known that people with disabilities experience poorer health and increased rates of health risks compared with the broader population, and may have lower levels of access to health promotion interventions [12,19,20,21,22,23,24]. Health promotion interventions in ICHI are in Section 3 “Interventions on the Environment” and Section 4 “Interventions on Health-related Behaviors”. Many health promotion intervention codes in ICHI can describe both interventions delivered to individuals and interventions delivered to broader groups or populations, e.g., “Education to influence tobacco use behaviors”. Some examples are given in Table 2.

Changes in various aspects of the social-ecological environment can lead to or support changes in health-related behaviours at an individual level and, to be most effective, complementary health promotion interventions should be implemented at different levels of the social-ecological system [25,26]. The ICHI extension code “System level at which intervention directed” can be used to describe the level of the social-ecological system at which the intervention is aimed (Box 1) [27]. This extension code is informed by the ecological model for health promotion of McLeroy and colleagues [28] and the ecological analysis scheme of Richard and colleagues [29]. It can distinguish between an intervention delivered directly to the person/people whose health it is seeking to influence and an intervention directed at a higher level of the social-ecological system; for example, training kindergarten teachers to facilitate children’s active play would be coded VEB PH ZZ “Training to influence physical activity behaviours”, with extension XGA4 to indicate that this intervention is intended to bring about change at the level of the organisation (kindergarten).

Box 1 **Extension code “System level at which intervention directed”.**

XGA1 **Individual**: the individual person for whom a health benefit is intended
XGA2 **Close interpersonal**: the close interpersonal environment of the individual including, e.g., immediate family members and informal carers
XGA3 **Extended interpersonal**: the wider interpersonal environment of the individual including, e.g., members of an informal social network
XGA4 **Organisation**: a grouping or association of people with a relatively formalised structure (e.g., school), including people who hold specific roles or positions within the organisation (e.g., teachers)
XGA5 **Community**: a geographical grouping of individuals (e.g., a district, city, neighbourhood).
XGA6 **Political system**: components and representatives of the political system of a given geographical entity

Within the broader context of health systems, health promotion interventions have a particular role to play in addressing health inequalities. However, depending on how they are designed and targeted, such interventions can be more effective for relatively advantaged groups within populations, and can fail to deliver health benefits for disadvantaged, vulnerable or marginalised groups, thus potentially exacerbating inequalities [30,31,32]. It is, therefore, important to know the extent to which health promotion interventions reach and are effective for vulnerable groups, such as people with disabilities, and ICHI clearly has a role to play here. While it does not capture all the dimensions of information relevant for understanding, evaluating and comparing health promotion interventions, ICHI will provide a standard basis for grouping interventions by type, using the three axes of Target, Action and Means. Additionally, the “system level” extension code will be of value in identifying interventions directed towards modifying social determinants operating at higher levels of the socio-ecological system, which may be more effective in achieving positive health outcomes for disadvantaged segments of the population.

### 3.3. An Example: Use of the Swedish National Classification of Social Care Interventions and Activities

The Swedish National Classification of Social Care Interventions and Activities (KSI) was developed during 2012–2014, and was based closely on ICHI. In the context of KSI “social care” includes interventions targeting activities and participation, environmental factors and health-related behaviours, plus a relatively small number of interventions targeting body functions. Over 270 providers in social care participated in a multi-step process with several meetings and workshops held over a period of about two years, to identify relevant targets, actions, means, and intervention codes based on ICHI for inclusion in KSI [33]. Social care providers also identified subsets of interventions within KSI applicable for the different areas of social care using a modified Delphi-method, to facilitate the use of relevant interventions in practice. 

Field trials have found KSI to be a very usable tool for describing social care interventions in the digital structured documentation system, and also for supporting practice. Social care workers record interventions planned and delivered, and use this information for local follow-up and monitoring within their organisation. Feedback from social care workers points to the benefits of KSI in providing a common language for use in the digital structured documentation system, and in fostering a common mind set about interventions in their work. In some social care practice areas, ICF and KSI have been used together to provide a common framework within which to record information about functioning, goal-setting, follow-up and, if needed, changes to interventions required in order to achieve client goals. This use of ICF and KSI together has been found to support evidence-based practice and quality improvement at both local level and national level [33,34,35].

## 4. Discussion 

ICHI promises to provide a broad-based and flexible classification to capture information on the full spectrum of health interventions that should be available to people with disabilities. It will provide a basis for collecting information concerning need for and equity of access to interventions. At an individual level, ICHI and ICF can be used together to record information on the process of goal-setting, evaluating intervention outcomes, and reviewing goals. The tri-axial structure of the classification provides a common model for thinking about and describing all types of health interventions. As an international standard that can function as a common language and framework for communication and data capture, ICHI will be a valuable tool for use in policy, research and practice. In particular, its adoption of the underpinning biopsychosocial model and its compatibility with the ICF will make it an important element of health information infrastructures in future. Use of ICD, ICF and ICHI together will provide a basis for monitoring health system effectiveness and improving quality of care.

Ten years in development, a beta version of ICHI is now available for testing. While intensive content development has been undertaken, much of the infrastructure required to support its implementation and consistent use is not yet in place. The ICHI Beta 2017 version does include a user guide, but this will need substantial expansion and refinement, and a classification index and education and training materials are yet to be developed. A program of beta testing is planned for 2018 and is expected to include mapping from existing interventions classifications, ICHI coding of health intervention data collections, coding of vignettes or standard cases, and ‘live’ coding of interventions delivered in particular practice settings. The purpose of this testing will be to identify what works well but also problems and shortcomings (e.g., content gaps, overlapping categories, ambiguities in code titles and definitions) so that these can be addressed to improve the classification before it is finalised [36]. Testing will also provide a basis for the development of education and training materials. The involvement of a diverse range of potential users in the testing program will be important so that the beta version of the classification can be appraised in terms of its ability to meet different user needs across a variety of countries and settings, including low-resource settings.

Following ICHI beta testing, implementation will become a major focus of activity for those in the WHO network of collaborating centres who have been involved in its development and are committed to seeing its potential realised. The history of ICF implementation over the 16-plus years since its endorsement by the World Health Assembly in 2001 shows that widespread uptake of a new classification does not happen instantly or automatically. Rather, it is a complex and gradual process involving exploration of its strengths and limitations in relation to a growing range of potential uses, collaboration and debate, and the progressive accumulation of a body of practice and experience in its use. Successive reviews of the literature have documented the steady development of practice and experience in ICF use in a wide variety of applications, as well as ongoing debate around some aspects of its operationalisation, including use of the qualifiers and the distinction between Activities and Participation [37,38,39]. Importantly, implementation of a new classification requires advocacy and commitment of resources. The costs and benefits of making changes to existing information infrastructures must be evaluated; in some cases, this will involve comparing ICHI against classifications currently in use.

## 5. Conclusions

ICHI is ambitious in its scope, spanning the full spectrum of health interventions and including types of intervention that have not previously been the focus of major classification effort (e.g., assistance with functioning and public health interventions). Its underlying tri-axial structure also makes it quite different in nature from the ICD and the classifications within each of the components of the ICF. No doubt experience generated through its early use will indicate need for future refinement, both of the classification itself and the accompanying guidelines and educational materials required to support its consistent use. In some respects, any international classification is always a work in progress: while it must provide a stable structure and basis for collecting data to support comparability over space and over time, there must also be scope for it to expand and evolve as required to serve the needs of its users. This is an inevitable tension that must be carefully negotiated by those who develop and maintain classifications. During the beta testing phase, input from those with an interest in the health of people with disabilities and health promotion more broadly is welcomed and will be essential in realising the potential of ICHI and maximising its utility for a broad range of applications.

Comments on the classification are welcome through the online platform: mitel.dimi.uniud.it/ichi/. (To provide comments, click ‘Sign in’ and create a free registered user account.)

## Figures and Tables

**Table 1:** Examples of ICHI interventions targeting body functions, activities and participation domains and environmental factors.

ICHI Intervention	Definition	ICHI Axis Categories
Title: Assisting and leading exercise for articulation functions Code: JUD PG ZZ	Supporting or guiding exercise focusing on functions of the production of speech sounds	Target: Articulation functions (ICF-code b320) Action: Assisting and leading exercise Means: Other and unspecified means
Title: Training in speaking Code: SFA PH ZZ	Teaching, enhancing or developing skills to produce words, phrases and longer passages in spoken messages with literal and implied meaning, through context-specific practice	Target: Speaking (ICF-code d330) Action: Training Means: Other and unspecified means
Title: Practical support to conversation Code: SGA RB ZZ	Providing practical assistance or guiding the person in starting, sustaining and ending a conversation and conversing with one or many people	Target: Conversation (ICF-code d350) Action: Practical support Means: Other and unspecified means
Title: Prescription of products and technology for communication Code: UAF TI ZZ	Instruction, direction or authoritative recommendation of equipment, products and technologies used by people in activities of sending and receiving information, including those adapted or specially designed, located in, on or near the person using them	Target: Products and technology for communication (ICF-code e125) Action: Prescription Means: Other and unspecified means

**Table 2:** Examples of health promotion intervention codes in ICHI.

ICHI Intervention	Definition	ICHI Axis Categories
Title: Environment modification to influence eating behaviours Code: VEA TM ZZ	Making physical changes to an indoor or outdoor environment to influence behaviour concerning patterns of eating, including frequency, quantity, and food choice	Target: Eating behaviours Action: Environment modification Means: Other and unspecified means
Title: Media campaign to influence screening behaviours Code: VDB PM QA	Delivering a planned series of communications using media to influence behaviours concerning patterns of use of health screening services	Target: Screening behaviours Action: Education Means: Media campaign
Title: Enforcement of legislation or regulations concerning outdoor air quality Code: UBM WI QE	Enforcing rules concerning air quality outside buildings or enclosed areas	Target: Outdoor air quality Action: Legislation or regulations, other Means: Enforcement
